# Effects of different pedagogical swimming teaching modes on teenagers' emotional adjustment ability and social adaptability

**DOI:** 10.3389/fpubh.2026.1829856

**Published:** 2026-06-10

**Authors:** Wanyun Feng

**Affiliations:** Tianjin Vocational College of Sports, Tianjin, China

**Keywords:** emotional regulation, pedagogical model, social adaptability, swimming teaching, teenagers

## Abstract

**Background:**

This study aims to reveal the influence of different pedagogical swimming teaching models on adolescents' emotional regulation and social adaptability.

**Methods:**

120 adolescents aged 12–16 years were randomly divided into comprehensive teaching group, cooperative learning group and traditional teaching group, 40 cases each, and the intervention was performed for 16 weeks. Adolescent Cognitive Emotion Regulation Questionnaire and Children's Social Adaptation Scale were used for evaluation, and combined with repeated measures analysis of variance and back-test.

**Results:**

After the intervention, the total score of emotion regulation and the dimension of cognitive re-evaluation in the cooperative learning group were significantly higher than those in the understanding group and the traditional group; The total scores of social adaptation, peer interaction, and collective integration to cooperative learning were the best. Comprehension teaching outperformed the traditional model in problem solving and emotional expression (*P* < 0.05). The traditional group showed no significant disadvantage in skill mastery, but the weakest improvement in emotional and social indicators. The intra-group time effect and the inter-group interaction effect were significant (*p* < 0.001). There was a significant positive correlation between the total score of adolescents' emotion regulation ability and the total score of social adaptability (r > 0.7, *p* < 0.001), and the slope of regression line in cooperative learning group was significantly higher than that in comprehension teaching group and traditional teaching group.

**Conclusion:**

The cooperative learning model has the most significant effect on promoting adolescents' emotional regulation and social adaptation, and provides structured exercise intervention programs for adolescents' mental health and social adaptation.

## 1 Introduction

Teenagers (12~18 years old) are in the critical period of “self-identity and role confusion” in Eriksson's personality development stage (1). Emotion Regulation Ability (ERA) and Social Adaptability (SA) are the core indicators of mental health and social development, which directly determine the quality of interpersonal interaction, psychological resilience and happiness in life (2). Emotional adjustment ability refers to the psychological ability of individuals to perceive and evaluate their own and others' emotions and adjust their emotional experience and expression through active strategies to meet environmental needs (3); Social adaptability is an individual's comprehensive ability to flexibly adjust his behavior, cognition and emotion, realize interpersonal interaction, collective integration and establish positive social relations in social scenes (4). At present, the mental health problems of adolescents in China are becoming younger and more prevalent. According to the relevant evaluation of the Organization for International Economic Cooperation and Development (OECD), the level of emotional anxiety of adolescent students is significantly higher than childhood, and the incidence of social estrangement and interpersonal sensitivity is increasing year by year, which has become an important problem restricting the all-round development of adolescents (5, 6).

As an important carrier of school mental health education, the scientific teaching mode of physical education directly affects the effectiveness of psychological intervention (7, 8). Among them, swimming teaching has irreplaceable advantages in emotional counseling and social training of teenagers because of the uniqueness of water environment (9). Swimming, as a whole-body aerobic exercise, can not only enhance teenagers' physique, but also ease the academic pressure and provide a safe scene for emotional venting. At the same time, the forms of double cooperation and group practice in swimming teaching also create natural conditions for social interaction (10). Pedagogical Model is Teaching Model (TM), which is called pedagogic model in English and abbreviated as PM. It refers to a systematic and standardized teaching framework built around teaching objectives, teaching processes and teaching methods under the guidance of certain educational theories. The core differences among the three teaching modes lie in the interactive structure, students' autonomy and the intensity of emotional support: the traditional guidance mode is teacher-centered and has low interaction; Understand that the teaching mode is problem-oriented and moderately autonomous; The cooperative learning model takes group task as the core, high emotion and social interaction, and this difference is the core theoretical framework for comparing the psychological benefits of different models in this study. The differences in teaching concepts and interactive forms of different Pedagogical models will have a differentiated impact on the psychological development of teenagers (11).

At present, in the field of swimming teaching at home and abroad, the mainstream pedagogic models mainly include traditional instructional pedagogic model (TIPM), understanding pedagogical model (UPM) and cooperative learning pedagogic model (CLPM) (12). The existing teaching practice mostly focuses on the acquisition of swimming skills and drowning prevention, ignoring the targeted cultivation of teenagers' emotional adjustment and social adaptability (13). Previous studies have shown that structured swimming teaching intervention can effectively improve teenagers' self-esteem and social integration, but there are still obvious gaps in the research on the differentiation effect of different pedagogical models (14). On the one hand, the existing research only discusses the psychological effect of a certain teaching mode, lacking the horizontal comparative analysis of three mainstream modes, and unable to clarify the advantages and disadvantages of different modes and applicable scenarios (15); On the other hand, most studies fail to specify the specific indicators of emotional adjustment (cognitive reappraisal, emotional repression) and social adaptability (peer interaction, collective integration), and fail to explain the internal mechanism of the influence of teaching mode on adolescent psychological development (16).

With the gradual emphasis on the psychological health value of physical education teaching under the background of the integration of physical education and education, it is required that physical education teaching realize the two-way empowerment of skill training and psychological development (17, 18). The purpose of this study is to clarify the differences in the influence of different pedagogical swimming teaching modes on teenagers' emotional adjustment ability and social adaptability, and to clarify its mechanism and intervention effect, which can not only enrich the cross-research results of physical education teaching psychology and teenagers' mental health education, but also provide scientific basis for swimming teaching reform, optimize the design of teaching mode, and make swimming teaching an important way to promote the coordinated development of teenagers' body and mind, which has important theoretical value and practical significance.

## 2 Methods

### 2.1 Research design and ethical approval

This study is a Randomized Controlled Trial, (RCT) approved by Tianjin University of Sport Ethics Committee (ethical batch number: TJUS2025-11). Teenagers aged 12-16 are taken as the research object, and three different pedagogical swimming teaching interventions are implemented through random grouping, with the intervention period of 16 weeks. Taking emotional adjustment ability and social adaptability as the main outcome indicators, and the mastery level of swimming skills as the secondary outcome indicators, combined with standardized scale evaluation and statistical analysis, the intervention effects of different teaching models were clarified. All research processes meet the ethical requirements of Helsinki Declaration, and all research subjects and their legal guardians have signed written informed consent.

### 2.2 Study objects

By stratified random sampling, adolescents aged 12–16 were selected as the research object, and the sample size was estimated according to GPower 3.1 software, with effect f = 0.25, α = 0.05, test efficiency (1–β) = 0.90, and the sample size of each group was at least 36 cases. Considering the follow-up dropout rate (expected 10%), the sample size was finally determined to be 120 cases. In this study, no subjects dropped out during the whole intervention process, and the number and reasons of dropping out were 0. All 120 subjects completed the intervention and various evaluations, and there was no lost visit. All the 120 subjects in this study completed the 16-week intervention and three time-point assessments, and there were no cases of dropping off or quitting, mainly due to strict attendance, one-on-one follow-up and standardized teaching guarantee.

#### 2.2.1 Inclusion criteria

Age 12~16 years old, regardless of sex; No swimming foundation (after pre-test, it is impossible to swim in any swimming stroke of 50 m independently); No history of mental illness or emotional disorder, no serious heart, lung, musculoskeletal diseases and contraindications for water sports; Did not participate in systematic physical training, psychological intervention or social skills training in the past 3 months; Can guarantee to participate in swimming teaching; The legal guardian informed consent and cooperated to complete the whole evaluation.

#### 2.2.2 Exclusion criteria

Those who cannot continue to participate in the trial due to injury, transfer and other reasons during the intervention period; Practice fraud in the evaluation process and fill in the questionnaire incompletely; Poor compliance; Those who participate in other activities (psychological counseling, competitive sports training) that may affect the outcome index during the intervention period.

#### 2.2.3 Grouping method

Using random number table method, 120 subjects were randomly divided into Understanding Pedagogical Model Group (UPMG) and Cooperative Learning Pedagogical Model Group (CLPMG) according to the ratio of 1:1:1 and the Traditional Instructional Pedagogical Model Group (TIPMG), 40 cases in each Group. The grouping was completed by independent statisticians who did not participate in teaching and evaluation, and the distribution was concealed by sealed envelope method. The researchers, teaching staff and evaluation staff did not know the grouping sequence, which was in line with CONSORT 2010 randomization and blind method specification. After grouping, the balance of age, gender, baseline emotional adjustment and social adaptability scores of the three groups was tested to ensure that the baselines between the groups were comparable. The baseline data of the three groups (age, sex, height, weight, total score of emotion regulation, and total score of social adaptability) were statistically tested, and the differences were not statistically significant (*P* > 0.05), which was comparable.

### 2.3 Intervention protocol

All three groups of subjects were taught swimming in the same standard swimming pool. The intervention intensity is uniformly set according to the percentage of the individual's maximum heart rate, and the target heart rate is 60%−75% of the individual's maximum heart rate (the maximum heart rate is calculated by the 220-year-old formula), and the group target interval of 110–140 beats/min is determined accordingly, and each training is monitored in real time through the heart rate bracelet; The training intensity and load are progressive in four stages: the first-fourth week is the adaptation period (the intensity is 40%−50%1RM, mainly for underwater adaptation and basic movements); The 5th−8th week is the promotion period (the intensity is 50%−60%1RM, and the decomposition action is intensified); 9–12 weeks is the consolidation period (intensity 60%−70%1RM, coherent action training); The 13th−16th week is a stable period (the intensity is maintained at 65%−70%1RM, 50m continuous swimming and endurance improvement), and each group is implemented according to the individual heart rate target to ensure that the intensity standards of the three groups are consistent and meet the individual physiological load. During the training process, the instructor uses the heart rate bracelet to check the heart rate of the trainees in real time, and records the heart rate data every 10 min. If it is lower than 110 beats/min, the action frequency and exercise density will be appropriately increased, and if it is higher than 140 beats/min, the low-intensity water relaxation adjustment will be arranged immediately to ensure that the whole process is stably maintained within the individual target heart rate interval; After class, the heart rate data are uniformly derived and checked to ensure the standardized implementation of intervention intensity.

Three teachers who have more than 5 years of swimming teaching experience and hold the national intermediate swimming coach certificate are the teaching teachers. Unified teaching mode training and standardized lesson preparation before teaching to ensure that the teaching process, content difficulty and teaching duration are highly consistent. Only the teaching mode has differentiated design. The intervention was expected to last for 16 weeks, three times a week for 90 min each time, and the cumulative intervention was 48 times. Each teaching includes three links: warm-up (15 min), core teaching (60 min) and relaxation and organization (15 min).

#### 2.3.1 Traditional instructional pedagogic model group

Traditional instructional pedagogic model (TIPM) group: The core is the standardized acquisition of skills. Through demonstration-imitation, the movement itself can relieve anxiety and provide basic emotional catharsis, but the social and autonomous emotional regulation training is insufficient. The traditional teaching mode of “teacher instruction-student imitation” is adopted, with swimming skill acquisition as the core goal. During the teaching process, teachers lead the whole process. Through demonstration (breaststroke ventilation, stroke action, etc.) and instruction issuance (“head-up ventilation, arm stroke synchronization,” etc.), students are required to imitate and practice uniformly on an individual basis, without setting up any form of group discussion, paying attention to standardized correction of actions, and lacking interactive communication and independent exploration links; The teaching content of skills is set according to the principle of gradual progress: Week 1~4 (basic adaptation): familiarity with water, holding breath, floating, and standing; Week 5~8 (basic movements): breaststroke arm stroke, leg pedal water decomposition training; Week 9~12 (coherent movement): breaststroke arm and leg movement coordination, ventilation exercise; Weeks 13–16 (skill consolidation): 50 m breaststroke continuous swim, movement optimization.

#### 2.3.2 Understanding pedagogical model group

Understanding pedagogical model (UPM) group: to stimulate independent thinking by questioning, to promote emotional expression by group discussion, to help teenagers actively identify emotions, to improve their problem-solving emotional adjustment ability, and to enhance basic social communication. Based on the theory of comprehensive teaching, the teaching mode of “problem-guided-self-explored-teacher-directed” is adopted, taking into account both skill acquisition and cognitive understanding. During the teaching process, teachers set up questions based on the difficulties of swimming skills (“Why is it easy to choke when ventilating? How to adjust body posture to reduce resistance?” etc.), and guided students to discuss in groups and try independently in the form of heterogeneous groups of 4 people. One group leader in each group was responsible for organizing speeches and records, and encouraged students to express confusion and experience in practice. Teachers only gave guidance when students encountered bottlenecks, and paid attention to cultivating students' independent thinking ability and emotional expression consciousness; The content of skills teaching is consistent with that of the traditional instruction group, but the only difference lies in the interactive form and guidance mode of teaching. Each core teaching link sets a 10-min group discussion time to encourage students to share their practice feelings and movement experiences.

#### 2.3.3 Cooperative learning pedagogic model group

Cooperative learning pedagogic model (CLPM) group: focusing on group tasks, mutual evaluation and mutual assistance, strengthening emotional perception, cognitive reappraisal and empathy, significantly improving social adaptability such as peer interaction and collective integration, and realizing the coordinated development of skills and psychology. Based on the theory of cooperative learning, the teaching model of “group cooperation-task-driven-mutual evaluation and mutual assistance” is adopted, with the dual goals of skill acquisition, emotional communication and social interaction. Each group of 40 students is further divided into 10 cooperative groups (4 people in each group). The group composition adopts heterogeneous grouping (taking into account age and gender differences), and the group division of labor (recorders, demonstrators, supervisors, sharers) is defined. During the teaching process, group tasks are set up (“completing the 50 m relay tour together, cooperating to discuss the optimization plan of breaststroke movements,” etc.), requiring members in the group to guide and encourage each other to solve problems in practice together; Teachers regularly organize group evaluation, encourage students to affirm others‘ merits, put forward suggestions for improvement, and pay attention to cultivating students' cooperative consciousness, communication ability and emotional empathy ability; The content of skills teaching is consistent with that of the first two groups. Each core teaching link is set up with 15 min group cooperation task time, and each time before the end of the course is set up with 5 min group sharing link to exchange emotional experience and cooperation feelings in the practice.

#### 2.3.4 Quality control

During the intervention period, an independent supervisor was arranged to follow up the whole process, record the teaching duration, teaching content and the implementation of interactive links of each group, and hold teaching summary meetings every week to correct teaching deviations in time. A punch-in system was adopted to record the attendance of students; telephone follow-up visits were conducted for absent students to understand the reasons for absence and urge them to make up the missed training in a timely manner. Regular teaching assessments were carried out for teachers to ensure the standardized and differentiated implementation of the three pedagogical teaching modes throughout the whole intervention.

### 2.4 Evaluation indicators and methods

Evaluation time points: before intervention (T0), 8 weeks of intervention (mid-period, T1), and 16 weeks of intervention (end-period, T2), a total of 3 time points. All evaluations were completed in the same environment and the same time period (9:00–11:00 am on weekends), and were carried out by evaluators who had undergone standardized training. The evaluators implemented single-blind grouping of the study subjects (did not know the group of the study subjects) to avoid evaluation bias.

#### 2.4.1 Main evaluation indicators

Emotional adjustment ability and social adaptability were evaluated by standardized scale. The Chinese version of the Adolescent Cognitive Emotion Adjustment Questionnaire (CERQ) has completed the cultural adjustment among the adolescents in China, eliminated the items that are not suitable for culture, and adjusted the expression, which is suitable for the adolescents aged 12–16 in China. Children's Social Adaptation Scale (CSAS) is revised in combination with social scenes of schools and families in China, which has good cultural validity and situational adaptability. The reliability and validity of the scale have been verified at home and abroad, and it is suitable for teenagers aged 12–16. Adolescent Cognitive Emotion Regulation Questionnaire (CERQ) Chinese version Cronbach's α = 0.88, test-retest reliability ICC = 0.82, structural validity χ^2^/df = 2.31, RMSEA = 0.06, CFI = 0.93; Chinese version of the Children's Social Adaptation Scale (CSAS) Cronbach's α = 0.87, test-retest reliability ICC = 0.80, structural validity χ^2^/df = 2.15, RMSEA = 0.05, CFI = 0.94, all of which meet the requirements of psychometrics.

##### 2.4.1.1 Emotion regulation ability

The Cognitive Emotion Regulation Questionnaire (CERQ) was used, which was compiled by Jermann et al. (19). The Chinese version was revised and used for Chinese adolescents. The internal consistency coefficient of the scale was Cronbach's α = 0.86~0.92, the test-retest reliability was r = 0.78~0.85, and the structural validity was good (χ^2^/df = 2.31, RMSEA = 0.06, CFI = 0.93). The scale has 36 items, including 9 dimensions such as cognitive re-evaluation, emotional repression, problem solving, self-blame, and rumination. It uses a 5-point scoring method (1 = almost never, 5 = almost always), and the total score ranges from 36 to 180 points. The higher the score, the stronger the ability of adolescents to regulate their emotions. Among them, the core observation dimensions were cognitive re-evaluation (4 items, ranging from 4 to 20 points), problem solving (4 items, ranging from 4 to 20 points), and emotional expression (4 items, ranging from 4 to 20 points), as the main analysis indicators. During the evaluation, the evaluators read out the instructions, and the research objects filled them in independently. After filling in, the questionnaire was collected on the spot, and the data was entered after checking that it was correct.

##### 2.4.1.2 Social adaptability

The Children's Social Adaptability Scale (CSAS) was used, which was revised by Chinese scholars and is suitable for adolescents aged 12~16 years (20). The internal consistency coefficient of the Scale was Cronbach's α = 0.83~0.90, the test-retest reliability was r = 0.76~0.84, and the structural validity was good (χ^2^/df = 2.15, RMSEA = 0.05, CFI = 0.94). There are 40 items in the scale, including four dimensions: peer interaction, collective integration, social self-confidence and emotional empathy. The 4-point scoring method is used (1 = completely inconsistent, 4 = completely consistent), and the total score range is 40~160 points. The higher the score, the stronger the social adaptability of adolescents; Among them, the core observation dimensions were peer interaction (10 items, ranging from 10 to 40 points) and collective integration (10 items, ranging from 10 to 40 points), as the main analysis indicators. The assessment process is consistent with CERQ to ensure the authenticity and completeness of the questionnaire.

#### 2.4.2 Secondary evaluation indicators

Level of swimming skill mastery to exclude interference from differences in skill acquisition on primary outcome measures. The national swimming grade standard (junior group) was used for evaluation. Two independent swimming coaches were jointly scored. The scoring consistency test was Kappa = 0.87 (*P* < 0.001). The evaluation indexes included: 50 m breaststroke swimming time (s) and movement standardization degree (full score of 10 points, divided into excellent 8~10 points, good 6~7 points, qualified 4~5 points and unqualified < 4 points). The average score of the two coaches was taken as the final score. The evaluation was carried out after the swimming teaching at the end of each time point (T0, T1, T2) to ensure that the evaluation environment was consistent with the teaching environment.

### 2.5 Statistical analysis

SPSS 26.0 software was used for statistical analysis. When the data meet the normal distribution (Shapiro-Wilk, *P* > 0.05) and the variance homogeneity (Levene, *P* > 0.05), repeated measurement analysis of variance is adopted. Robust variance analysis is used when normal distribution or variance homogeneity is not satisfied; The measurement data are expressed as mean standard deviation (x ± s), the comparison between groups is made by one-way ANOVA, and the repeated measurement data is made by ANOVA, and the main effect, time main effect, interaction effect, F value, *P* value, Partial η^2^ and 95% CI; between groups are reported. χ test was used for counting data, and paired *t* test was used for comparison before and after the group. Pearson correlation analysis was used to analyze the correlation, and all statistical preconditions and inspection methods were reported in a unified way, with the inspection level α = 0.05.

## 3 Results

### 3.1 Baseline data on demographic and basic swimming skills of the three groups of subjects

There was no significant difference in baseline scores of each dimension of emotion regulation and social adaptability before intervention between the three groups (*P* > 0.05), which was comparable. The three groups of subjects were compared on demographic indexes such as age, gender, height and weight, as well as basic skill indexes such as swimming technique enrollment rate and basic movement standard score. Through one-way analysis of variance (measured data) and chi-square test (counted data), the results showed no significant difference between the indicators (*P* > 0.05), and the baseline characteristics were balanced and consistent with good comparability. See Table 1.

**Table 1:** Comparison of demographic characteristics and basic swimming skills among three groups of teenagers (x ± s, *n*/%, *n* = 40/group).

Index	CL group	UM group	TM group	Test value	*P*-value
Age (years)	14.25 ± 1.32	14.18 ± 1.29	14.32 ± 1.35	F = 0.215	0.807
Gender (M/F, *n*)	21/19	20/20	22/18	χ^2^ = 0.200	0.904
Percentage of men (%)	52.5	50	55	χ^2^ = 0.200	0.904
Height (cm)	162.35 ± 7.58	161.89 ± 7.42	162.68 ± 7.65	F = 0.157	0.855
Body weight (kg)	52.18 ± 8.36	51.75 ± 8.29	52.45 ± 8.42	F = 0.124	0.883
Swimming skills entry compliance rate (*n*, %)	33 (82.50)	32 (80.00)	34 (85.00)	χ^2^ = 0.587	0.746
Normative score of basic swimming movements (points)	65.32 ± 5.17	64.89 ± 5.23	66.05 ± 5.09	F = 0.817	0.444

CL Group: cooperative learning pedagogic model (CLPM) group; UM Group: understanding pedagogical model (UPM) group; TM Group: traditional instructional pedagogic model (TIPM) group. The measurement data were analyzed by one-way ANOVA, and the counting data were tested by χ test. *P* > 0.05 means there is no significant difference between the two groups.

### 3.2 Intervention effect of different pedagogical modes of swimming teaching on adolescents' emotion regulation ability

After 16 weeks of intervention, all dimensions and total scores of emotion regulation ability of adolescents in the three groups were significantly improved compared with those before intervention (*P* < 0.001 in the group); Comparison between groups showed that the scores of each index in cooperative learning group were the highest, which was significantly higher than that in understanding teaching group and traditional teaching group (*P* < 0.001); The scores of problem solving and emotion expression dimensions in the comprehension teaching group were significantly higher than those in the traditional teaching group (*P* < 0.05); Between groups, *F* = 3.214~78.452, *P* < 0.05, the interaction effect was significant. See Table 2 for specific data.

**Table 2:** Comparison of scores of emotion regulation ability in three groups of adolescents before and after 16 weeks of intervention (x ± s, *n* = 40/group).

Dimension	Group	Pre-intervention	Post-intervention	Intra-group F-value	Intra-group *P*-value	Intergroup F-values	*P*-value between groups
Cognitive reevaluation	CL group	15.64 ± 2.89	18.58 ± 3.22	128.671	< 0.001	62.385	< 0.001
	UM group	14.98 ± 2.76	17.11 ± 2.94	89.452	< 0.001		
	TM group	14.52 ± 2.81	16.82 ± 3.01	36.293	< 0.001		
Problem solving	CL group	14.31 ± 2.45	18.89 ± 2.73	97.562	< 0.001	28.573	< 0.001
	UM group	13.87 ± 2.39	17.65 ± 2.57	72.341	< 0.001		
	TM group	13.54 ± 2.42	15.33 ± 2.41	32.684	< 0.001		
Emotional expression	CL group	11.26 ± 2.01	16.95 ± 2.26	85.431	< 0.001	24.392	< 0.001
	UM group	10.93 ± 1.98	15.22 ± 2.13	65.792	< 0.001		
	TM group	10.68 ± 2.03	13.17 ± 2.08	29.873	< 0.001		
Total score	CL group	51.06 ± 4.92	62.32 ± 5.16	156.892	< 0.001	78.452	< 0.001
	UM group	49.50 ± 4.78	58.47 ± 4.89	112.673	< 0.001		
	TM group	48.39 ± 4.85	53.61 ± 5.33	45.921	< 0.001		

CL Group: cooperative learning pedagogic model (CLPM) group; UM Group: understanding pedagogical model (UPM) group; TM Group: traditional instructional pedagogic model (TIPM) group. Repeated measurement analysis of variance was used for intra-group comparison, and one-way analysis of variance was used for inter-group comparison. *P* < 0.05 is statistically significant.

### 3.3 Intervention effects of different pedagogical modes of swimming teaching on adolescents' social adaptability

After 16 weeks of intervention, all dimensions and total scores of social adaptability of adolescents in the three groups were significantly improved compared with those before intervention (*P* < 0.001 within the group); Comparison between groups showed that the cooperative learning group had the highest scores in peer interaction and collective integration dimensions, which was significantly higher than the other two groups (*P* < 0.001); The scores of each index in the comprehension teaching group were significantly higher than those in the traditional teaching group (*P* < 0.05); Between groups, F = 4.265~65.893, *P* < 0.05, the interaction effect was significant. See Table 3 for specific data.

**Table 3:** Comparison of social adaptability scores of three groups of adolescents before and after 16 weeks of intervention (x ± s, *n* = 40/group).

Dimension	Group	Pre-intervention	Post-intervention	Intra-group F-value	Intra-group *P*-value	Intergroup F-values	*P*-value between groups
Peer interaction	CL group	16.32 ± 3.15	24.16 ± 3.51	115.782	< 0.001	59.374	< 0.001
	UM group	15.89 ± 3.08	20.89 ± 3.26	78.451	< 0.001
	TM group	15.47 ± 3.12	17.54 ± 3.19	30.263	< 0.001
Collective integration	CL group	13.56 ± 2.65	21.33 ± 2.87	102.453	< 0.001	51.632	< 0.001
	UM group	13.12 ± 2.58	18.65 ± 2.73	69.871	< 0.001
	TM group	12.78 ± 2.62	15.82 ± 2.68	28.544	< 0.001
Adaptation of social rules	CL group	12.45 ± 2.23	16.98 ± 2.45	56.782	< 0.001	18.953	< 0.001
	UM Group	12.13 ± 2.19	15.82 ± 2.37	48.321	< 0.001
	TM group	11.89 ± 2.21	13.96 ± 2.41	22.653	< 0.01
Environmental adaptation	CL group	7.26 ± 1.72	9.98 ± 1.89	35.431	< 0.001	6.892	< 0.01
	UM group	7.03 ± 1.68	9.02 ± 1.76	29.672	< 0.001
	TM group	6.85 ± 1.70	8.92 ± 1.83	25.314	< 0.001
Movement collaboration	CL group	8.15 ± 1.85	10.00 ± 1.98	24.562	< 0.01	4.265	< 0.05
	UM group	8.02 ± 1.81	9.65 ± 1.92	21.343	< 0.01
	TM group	7.95 ± 1.83	9.45 ± 1.95	19.874	< 0.01
Total score	CL group	57.74 ± 5.62	72.45 ± 6.03	132.563	< 0.001	65.893	< 0.001
	UM group	56.21 ± 5.48	65.38 ± 5.76	95.782	< 0.001
	TM group	54.94 ± 5.56	58.24 ± 5.91	38.651	< 0.001

CL Group: cooperative learning pedagogic model (CLPM) group; UM Group: understanding pedagogical model (UPM) group; TM Group: traditional instructional pedagogic model (TIPM) group. Repeated measurement analysis of variance was used for intra-group comparison, and one-way analysis of variance was used for inter-group comparison. P < 0.05 is statistically significant.

**Figure 1:**
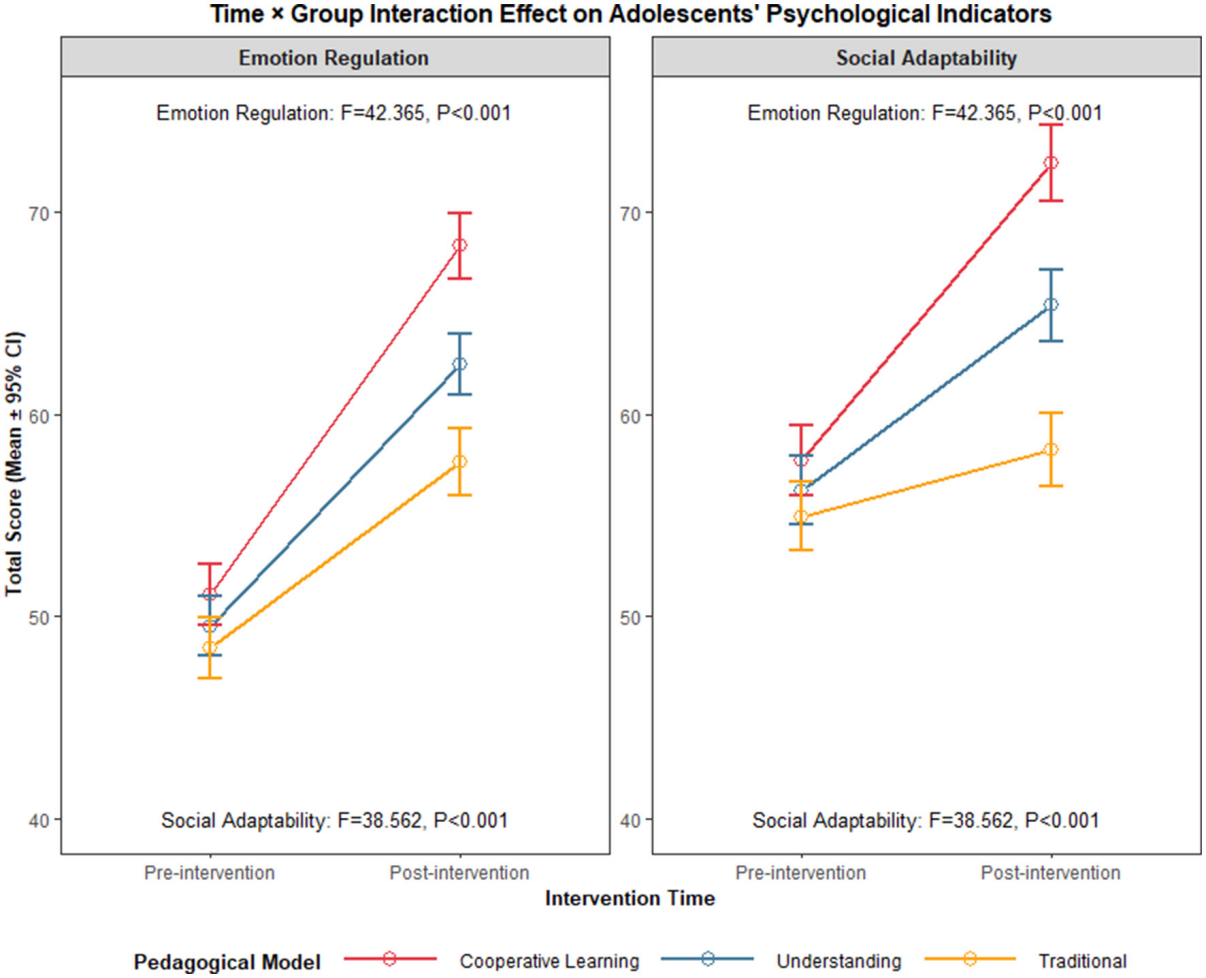
Total score of emotion regulation and total score of social adaptation of three groups of adolescents before and after intervention × group interaction effect plot (marked on the F and *P* value plots).

There was no significant baseline difference in emotion regulation and social adaptability scores between the three groups before the intervention. After 16 weeks of different mode swimming teaching intervention, the scores of the three groups were significantly improved and there was a very significant time × group interaction effect (Figure 1). Among them, the scores of emotion regulation (F = 42.365, *P* < 0.001) and social adaptability (F = 38.562, *P* < 0.001) in the cooperative learning group were significantly higher than those in the comprehensive teaching group and the traditional instruction group, and the comprehensive teaching group was better than the traditional instruction group.

### 3.4 Intervention effects of different pedagogical models of swimming teaching on adolescents' swimming skills mastery

**Figure 2:**
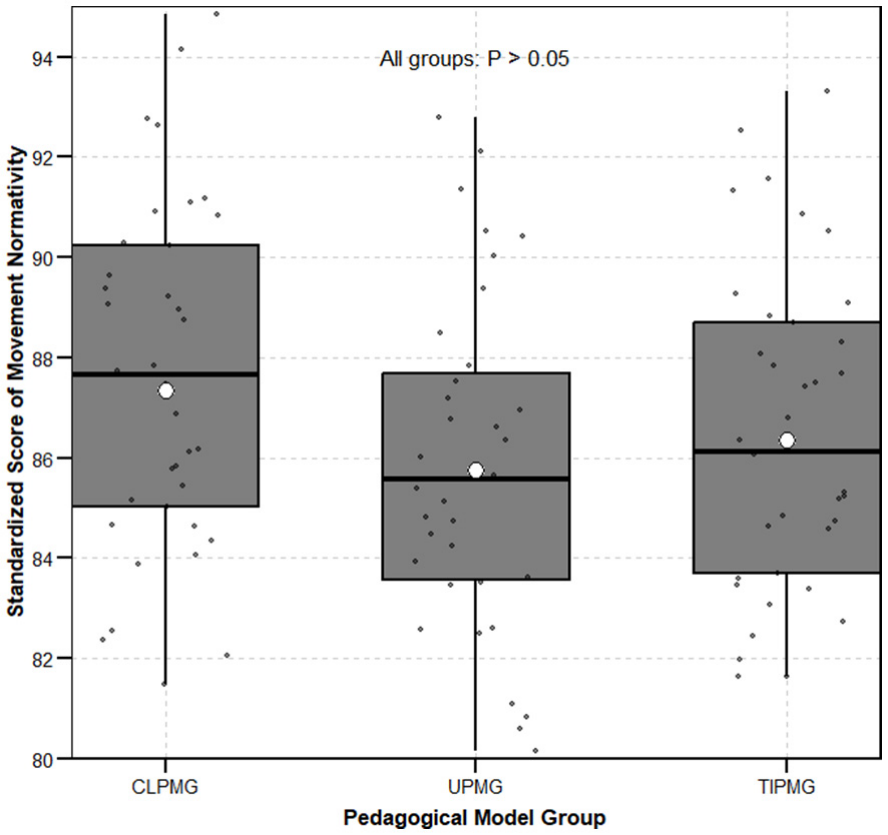
Box plot of the distribution of normative scores of swimming movements in three groups of adolescents during 16 weeks of intervention. The abscissa is the group, and the ordinate is the action normative score (points); The box is the quartile distance (Q1–Q3), the midline is the median, and the whisker line is the minimum value to the maximum value; There was no statistically significant difference between the groups (*P* > 0.05).

There was no significant difference in swimming skill achievement rate and movement normative score between the three groups (*P* > 0.05). After the intervention, the skill achievement rate and standard movement score were used as the core indexes to evaluate the swimming skill mastery effect of the three groups of adolescents. One-way analysis of variance (measured data) and chi-square test (counted data) showed no significant difference between the three groups (*P* > 0.05). See Table 4 for specific data. In the cooperative learning group, the skill achievement rate was 97.50%, and the standard action score was 87.15 ± 4.49; in the comprehensive teaching group, the achievement rate was 92.50%, and the action score was 85.78 ± 4.62; in the traditional instruction group, the success rate was 95.00%, and the action score was 86.32 ± 4.57. F-value = 0.932 (movement norm score), χ^2^ = 1.157 (skill achievement rate), and *p*-value among groups were all > 0.400 (Figure 2). The median and quartile ranges of achievements in the three groups were highly coincident, with no significant differences between the groups.

**Table 4:** Comparison of swimming skill achievement rate and action standardization score of three groups of teenagers after 16 weeks of intervention (x ± s, *n*/%, *n* = 40/group).

Index	CL group (CLPMG)	UM group (UPMG)	TM group (TIPMG)	Test value	*P*-value
Skills compliance rate (*n*, %)	39 (97.50)	37 (92.50)	38 (95.00)	χ ^2^= 1.157	0.56
Action normative score (Points)	87.15 ± 4.49	85.78 ± 4.62	86.32 ± 4.57	F = 0.932	0.4

CL Group: cooperative learning pedagogic model (CLPM) group; UM Group: understanding pedagogical model (UPM) group; TM Group: traditional instructional pedagogic model (TIPM) group. Normative action score out of 10 points; The measurement data were analyzed by one-way ANOVA, and the counting data were tested by χ test. *P* > 0.05 means there is no significant difference between the two groups.

### 3.5 Moderating effects of gender and age on the intervention effect of teaching model

Results from repeated measures ANOVA showed that gender and age did not significantly moderate the intervention effects of the three teaching models on emotion regulation and social adaptation (all *p* > 0.05) (Table 5).

**Table 5:** Analysis of the moderating effect of gender and age on the intervention effect of three teaching models (repeated measurement variance analysis).

Moderation variable	Dependent variable	Intergroup main effect F value	Principal effect *P* value between groups	Time × gender interaction f-value	Time × gender interaction *p*-value	Time × age interaction f value	Time × age interaction *p*-value
Gender	Total emotion regulation score	1.247	0.266	0.892	0.411	-	-
	Total social adaptability score	1.083	0.34	0.765	0.466	-	-
Age group	Total emotion regulation score	2.156	0.144	-	-	1.127	0.326
	Total social adaptability score	1.879	0.172	-	-	0.984	0.376

The dependent variables are the total score of emotion regulation and the total score of social adaptability; The moderating variables are gender and age group; F value is the main effect or interaction effect between groups; *P* > 0.05 means that the regulatory effect is not statistically significant.

### 3.6 Correlation analysis of adolescents' emotion regulation ability and social adaptability under different teaching models of swimming teaching

**Figure 3:**
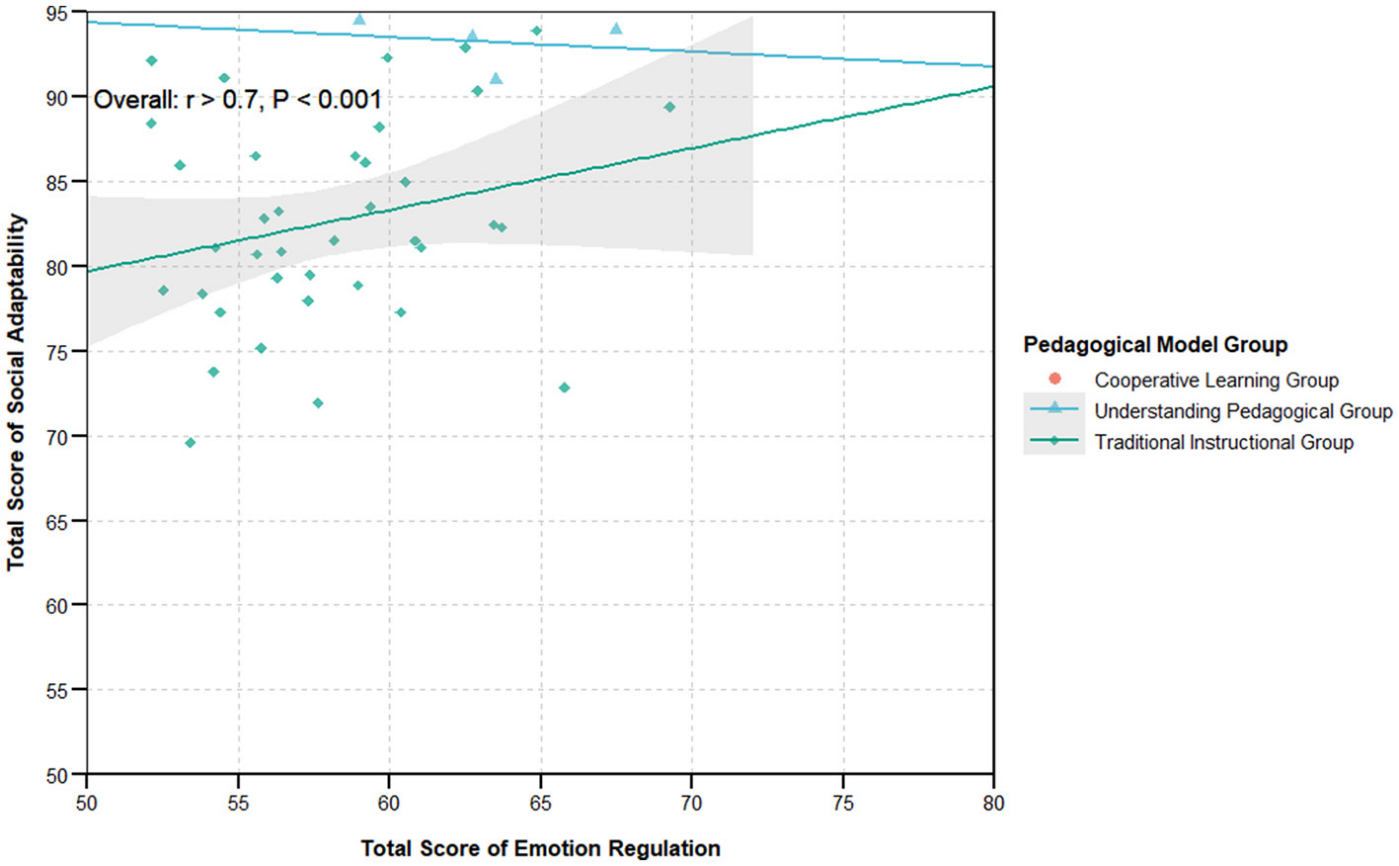
Scatter plot of correlation between total score of emotion regulation and total score of social adaptation of three groups of adolescents (labeled on r and *P* value plots).

After 16 weeks of swimming teaching intervention with different teaching models, the total scores of emotion regulation ability and social adaptability of adolescents were significantly strongly positive correlated (r > 0.7, *P* < 0.001), and the slope of regression line in cooperative learning group was significantly higher than that in comprehensive teaching group and traditional instruction group. The improvement of emotion regulation ability in this group had a stronger predictive effect on the improvement of social adaptability, and all three groups showed obvious positive correlation distribution characteristics. See Figure 3.

## 4 Discussion

Through a randomized controlled trial, this study systematically explored the differentiated effects of three traditional swimming teaching models, guided, comprehensive and cooperative learning, on the emotional regulation ability and social adaptability of adolescents aged 12–16 years, and verified the synergistic development characteristics of adolescents' emotional and psychosocial indicators under structured swimming intervention (21). The results show that the cooperative learning mode has a more significant positive effect on promoting adolescents' emotion regulation and social adaptability, the understanding teaching mode has a moderate promotion effect, and the traditional guidance mode has a relatively limited promotion effect. Swimming teaching can gradually shift from single skill orientation to coordinated development of body and mind, and prioritize the teaching design based on cooperation and understanding, so as to provide structured exercise intervention reference scheme for adolescents' mental health and social adaptation. This conclusion provides a direct empirical basis for the transformation of swimming teaching from skill-oriented to body-mind synergistic development oriented under the background of integration of physical education and education, and also provides a new idea for the design of structured exercise intervention scheme of adolescent mental health education (22, 23).

The outstanding advantages of cooperative learning models in improving adolescents' emotion regulation ability are concentrated in the significant improvement in the core dimension of cognitive reevaluation (24). The cooperative learning model adopts heterogeneous grouping, task-driven and mutual evaluation and mutual assistance mechanisms to construct high-frequency and positive social interaction scenes for teenagers: in swimming cooperative tasks such as 50-meter relay swimming and joint research on movement optimization schemes, teenagers need to actively perceive the emotional state of team members, adjust their emotional expression and behavioral response through cognitive re-evaluation, and coordinate interpersonal differences during practice. This immersive emotional practice experience allows adolescents to repeatedly exercise their cognitive re-evaluation ability, which is highly consistent with the conclusion of existing research that social interaction practices are the core path to improve adolescents' emotional regulation strategies (25). At the same time, the exclusive emotion sharing link set up by the cooperative learning mode provides a safe channel for teenagers to express their emotions, so that their problem solving and emotion expression ability can be improved simultaneously, and finally promote the total score of emotion regulation to reach the optimal level of three groups (26). Although the understanding teaching mode cultivates students' consciousness of self-thinking and emotion expression through question guidance and group discussion, its interaction depth and frequency are not as good as cooperative learning mode, so the improvement effect of each dimension of emotion regulation is second; The traditional teaching model with teachers' one-way dominance as the core lacks students' autonomous exploration and emotional interaction links, and can only achieve a small improvement in psychological indexes through the emotional catharsis effect of swimming itself, which also explains that its emotion regulation score is significantly lower than the results of the first two groups. The results of this study suggest that the cooperative learning model may promote the ability of cognitive reevaluation through high-frequency positive social interaction, and this mechanism needs to be further verified by longitudinal tracking and brain science research.

In terms of social adaptability training, the advantages of cooperative learning model are reflected in the significant improvement of two core dimensions of peer interaction and collective integration. A clear division of roles (recorder, demonstrator, etc.) in the cooperative learning process enables adolescents to break through the barriers of individual learning and establish positive peer relationships in mutual guidance and encouragement of swimming skills practice; The sense of group honor formed in the process of completing the collective task effectively strengthens its sense of collective integration and belonging. It is worth noting that there was no significant difference between the cooperative learning group and the integrated teaching group in the dimensions of social rule adaptation and motor cooperation, and both groups were significantly superior to the traditional teaching group. This result suggests that student-centered interactive instructional design is key to improving adolescents' social adaptability, whether task-driven cooperative learning or problem-oriented integrated instruction, enabling students to master social rules and collaborative skills in interaction (27). However, the traditional teaching mode, one-way teaching mode, blocks this training path. This discovery further refines the internal mechanism of swimming teaching mode affecting adolescents' social adaptability, and clarifies the core design direction for subsequent teaching mode optimization. This study speculates that the role division and mutual evaluation and mutual assistance link in cooperative learning are helpful for peer interaction and collective integration, and the relevant path still needs to be directly verified by mediation effect analysis.

This study found that there was a significant strong positive correlation between adolescents' emotion regulation ability and total social adaptation score (r > 0.7, *P* < 0.001), verifying the intrinsic correlation between adolescents' emotional development and social development (28). The slope of the regression line in the cooperative learning group is higher, which indicates that the model can strengthen the positive prediction effect of emotion regulation ability on social adaptability. In essence, the cooperative learning model simultaneously promotes the improvement of adolescents' emotional perception, empathy and communication ability. The improvement of emotion regulation ability can help adolescents better deal with interpersonal conflicts in social interaction, reduce social anxiety and alienation, and then form a positive cycle of improvement of emotion regulation-smooth social interaction-improvement of social adaptability (29). On the other hand, due to the lack of effective emotional and social interactions, the two psychological indicators of adolescents improved slowly, and the correlation between the two was significantly weaker than that of the first two groups, indicating that the role of single skill training on the overall development of adolescents' psychology was limited (30). This study confirms that there is no significant difference in the mastery of swimming skills between the three teaching models, which indicates that integrating the elements of cooperation and inquiry in swimming teaching can realize the synchronous training of psychological ability without affecting the skill acquisition, and eliminates the core concerns for the reform and innovation of swimming teaching.

There are still some limitations in this study. This study only selects breaststroke teaching, does not involve freestyle, backstroke and other swimming strokes, the teaching content is single; The confounding factors such as coaches' teaching style, family parenting style and school peer environment were not controlled; The sample was limited to adolescents aged 12–16 years without basic swimming, and the extrapolation was limited; Long-term follow-up was not performed and the durability of psychological benefits was not verified; No mediation/modulation analysis was performed and the mechanism of action was only indirect speculation. The teachers were three intermediate coaches, and the influence of individual differences in teaching style on the intervention effect was not considered; The study did not control the confounding factors such as the family environment and school social climate of adolescents. Future research plans will extend to multiple stroke comparisons to verify model generalizability, incorporate teacher variables across different teaching styles to quantify teacher impact, and control for confounders such as home and school environments to construct more accurate ecological intervention models, while extending the follow-up period to assess the long-term durability of psychological benefits.

## 5 Conclusion

In summary, the cooperative learning mode has a more significant positive effect on promoting adolescents' emotional regulation and social adaptability, while the understanding teaching mode has a moderate promotion effect, while the traditional guidance mode has a relatively limited promotion effect. The practice of swimming teaching should abandon the single idea of skill-oriented teaching, take cooperative learning and comprehensive teaching as the main teaching design, and build a teaching system of body and mind synergistic development with skill acquisition, emotional experience and social communication as the core. In the teaching process, through heterogeneous grouping, task-driven, emotional sharing and other links, students' social interaction and emotional practice experience are enriched, so that swimming teaching not only becomes a way for teenagers to master sports skills and enhance physical fitness, but also becomes a structured sports intervention carrier to improve teenagers' mental health level and cultivate social adaptability, so as to truly realize the educational value of physical education under the background of integration of sports and education.

## Data Availability

The original contributions presented in the study are included in the article/supplementary material, further inquiries can be directed to the corresponding author.
